# Nationwide monitoring of end-of-life care via the Sentinel Network of General Practitioners in Belgium: the research protocol of the SENTI-MELC study

**DOI:** 10.1186/1472-684X-6-6

**Published:** 2007-10-08

**Authors:** Lieve Van den Block, Viviane Van Casteren, Reginald Deschepper, Nathalie Bossuyt, Katrien Drieskens, Sabien Bauwens, Johan Bilsen, Luc Deliens

**Affiliations:** 1 End-of-Life Care Research Group, Vrije Universiteit Brussel, Belgium; 2 Scientific Institute of Public Health, Unit of Epidemiology, Brussels, Belgium; 3 Centre for Oncology, University Hospital Brussels, Belgium; 4 Bioethics Institute, Ghent University, Ghent, Belgium; 5 Department of Public and Occupational Health, and EMGO Institute, VU University Medical Centre, Amsterdam, The Netherlands

## Abstract

**Background:**

End-of-life care has become an issue of great clinical and public health concern. From analyses of official death certificates, we have societal knowledge on how many people die, at what age, where and from what causes. However, we know little about how people are dying. There is a lack of population-based and nationwide data that evaluate and monitor the circumstances of death and the care received in the final months of life. The present study was designed to describe the places of end-of-life care and care transitions, the caregivers involved in patient care and the actual treatments and care provided to dying patients in Belgium. The patient, residence and healthcare characteristics associated with these aspects of end-of-life care provision will also be studied. In this report, the protocol of the study is outlined.

**Methods/Design:**

We designed a nationwide mortality follow-back study with data collection in 2005 and 2006, via the nationwide Belgian Sentinel Network of General Practitioners (GPs) i.e. an existing epidemiological surveillance system representative of all GPs in Belgium, covering 1.75% of the total Belgian population. All GPs were asked to report weekly, on a standardized registration form, every patient (>1 year) in their practice who had died, and to identify patients who had died "non-suddenly." The last three months of these patients' lives were surveyed retrospectively. Several quality control measures were used to ensure data of high scientific quality.

**Discussion:**

In 2005 and 2006, respectively 1385 and 1305 deaths were identified of which 66% and 63% died non-suddenly. The first results are expected in 2007. Via this study, we will build a descriptive epidemiological database on end-of-life care provision in Belgium, which might serve as baseline measurement to monitor end-of-life care over time. The study will inform medical practice as well as healthcare authorities in setting up an end-of-life care policy. We publish the protocol here to inform others, in particular countries with analogue GP surveillance networks, on the possibilities of performing end-of-life care research. A preliminary analysis of the possible strengths, weaknesses and opportunities of our research is outlined.

## Background

End-of-life care has become an issue of great clinical and public health importance and concern. Developments contributing to this are the growing number of elderly people, the increasingly chronic nature of dying in developed societies, the accompanying rise in health care costs, and the growing recognition that end-of-life care is less than optimal in many countries [1-4].

However, there is a lack of population-based and nationwide data that evaluate and monitor how and in what circumstances people are dying and what care they are receiving in the final months of life [3-6]. From the analysis of mortality statistics based on official death certification, we have learned how many people die, at what age, from what causes, and where e.g. [7], but no such systematic nationwide data are being gathered concerning the (quality of) end-of-life care. Several efforts have been made to explore data relevant for end-of-life care in existing large-scale databases such as disease registries or healthcare billing data, and newly developed population-based surveys have shed light on important aspects of quality in end-of-life care, mainly in the UK and US [8-14]. But several basic population-based descriptions of how end-of-life care is organised for all dying patients in a whole country, are lacking on an international level [1-6]. Important gaps in knowledge include: (1) who takes care of the dying, how often are specialized multidisciplinary palliative care teams involved and which factors affect their involvement; (2) how much time is spent in different care settings at the end of life and how often are patients transferred between care settings at the end of life; (3) what type of care is provided to patients at the end of life: what are the main goals of medical treatment, and how often is patient care focused at palliative care as recommended by the World Health Organisation [1,2]?

Almost all earlier studies are restricted to specific populations of patients (such as cancer or elderly patients) or to specific settings (such as nursing homes, hospices or hospitals), have small sample sizes or focused on a specific aspect of end-of-life care provision, thus providing only a limited view on how people are dying in a society [11,15-26].

The absence of this basic epidemiological information hampers the ability to develop an effective public health policy on end-of-life care. Gathering epidemiological data on the places of care, the caregivers involved, and the actual care given is pivotal to the rational planning, organisation, and implementation of healthcare services for the dying and an important first step towards improving end-of-life care.

Specifically for Belgium, end-of-life care research has primarily been focused on euthanasia and other end-of-life decisions [27,28], while further empirical knowledge on the provision of end-of-life care is scarce. This contrasts sharply with the societal and political attention to "end-of-life-issues," considering its recently approved laws on palliative care, patient rights and euthanasia (2002) [29-31].

This "SENTI-MELC study" (Sentinel Network study Monitoring End-of-Life Care) is the first nationwide, population-based study on end-of-life care provision in Belgium. For the purpose of measuring across patient populations and care settings, we explored the possibilities of using an existing surveillance network, namely the Belgian Sentinel Network of General Practitioners.

### Aims of the study

The over-riding objectives of this SENTI-MELC study are:

1) To map out the places of care and death, and the care setting trajectory of patients at the end of life

2) To describe the caregivers involved in end-of-life care, and the actual treatments and care provided at the end of life; and more specifically to study the role of the general practitioner and of specialized multidisciplinary palliative care services

3) To study the patient, residence, and healthcare characteristics associated with places of care, caregivers, and end-of-life care provided.

The objective of this report is to present the protocol of the study used for the data collection in 2005 and 2006. We hope that our experience in Belgium will be useful to others, in particular to countries with analogue surveillance networks of general practitioners [32,33] who wish to initiate end-of-life care research into their registrations. The described methodology will also serve as detailed reference for the method section of future SENTI-MELC publications as results from the study emerge.

## Methods/Design

Because of the problems with prognosticating who is dying, it is difficult, if not impossible, to obtain information from a representative sample of dying patients in prospective end-of-life care research [34,35]. Therefore, a retrospective nationwide mortality follow-back survey, with data collection shortly after the patient had died, was chosen for this study.

### Observational unit

General practice is highly accessible in Belgium: almost all of the population (95%), including care home residents, have a regular general practitioner (GP) who they consult regularly [36]. Hence, GPs can provide a good public health perspective on end-of-life care and dying in the country and it would be possible to generate a population-based sample of deaths via GPs. Therefore, we decided to work with a representative sample of GPs in Belgium.

A Sentinel Network of General Practitioners is a network of practices or community based physicians who monitor one, several or an exhaustive list of health problems on a regular or continuing basis. The information from these practices is used to monitor the health of the entire population [32,37]. The Belgian Sentinel Network of GPs is a nationwide network, operational since 1979, and has proved to be a reliable surveillance system for a wide variety of health-related epidemiological data e.g. on diabetes, stroke, cancer, accidents [33,38-43].

The GPs in the Network collect data via continuous and weekly paper-and-pencil registrations using a standardized registration form. Each registration programme lasts minimally one year. Each year, about 8 different themes are surveyed. The general objectives of a Sentinel Network of GPs are: to evaluate public health problems and their importance within the general population; to continuously observe certain health problems over time for example to study the impact of prevention campaigns; and to study the management and follow-up of health problems by the general practitioners. Recorded data must concern an important health problem not subject to surveillance of another system, unless the Sentinel Network provides complementary information to this end. The participation of the GPs is voluntary. Feedback is regularly distributed to the participants, concerned authorities, the medical press, scientific associations and interested individuals. The turnover of the GPs, from year to year, is low, which contributes to the collection of data of high scientific quality. Also, only regularly participating GPs (i.e. who register at least 26 weeks per year) are included for data analyses. The Sentinel Network is funded by the regional governments of Belgium and co-ordinated by the Scientific Institute of Public Health [44].

In 2005, the first year of this study, the Network consisted of 181 regularly participating GP practices (205 GPs), who were representative of all 10,578 GPs in Belgium in terms of age, gender and geographical distribution, and also of the GPs in the Northern (Dutch-speaking) and Southern (French-speaking) regions of Belgium separately. The Network covered 1.75% of the total Belgian population [40,44].

### Study population

The unit of measurement in this study was the death case. Primary inclusion criteria were:

- every patient, part of the practice of the GP, who had died (certified deaths and deaths of which they were informed afterwards)

- aged one year or older

In order to focus this study on care delivered at the end of life or on dying patients (i.e. patients who were theoretically able to receive care in the terminal phase of life) we additionally excluded all deaths that had occurred "suddenly and totally unexpectedly [28,45]."

### Retrospective data collection procedure

For the purpose of this study, the GPs registered deaths on a weekly standardized registration form, during 2 consecutive years (2005–2006) from January 1^st ^until December 31^st^. To shorten the time between death and registration – hence preventing recall bias as much as possible – the physicians were instructed to register all deaths, immediately after being informed about the patient's death. GPs use patient records and information coming from hospital physicians as much as possible when filling in the forms. To control for possible non-response (e.g. GPs who forgot to report one of their deaths), all GPs received a summary of all reported deaths after each year of registration. GPs were asked to verify all these reported deaths and indicate if there were deaths that were not yet reported. If this was the case, we asked about basic demographic information (age, sex, place and cause of death) of the unreported deaths.

### Definition of concepts

In this study, the terminal phase under consideration was defined as the last three months of life of patients that died non-suddenly [34,46-48].

*Transitions between end-of-life care settings *were defined as moves or changes in location of care, during the last three months of life. Home (or with relatives, in service flats), care home (elderly/nursing home), hospital and (inpatient) palliative care unit, were differentiated. Care setting trajectories are a combination of moves between different settings.

*Caregivers *involved in patient care were subdivided into informal caregivers (e.g. partner, child, sibling, friends) and formal caregivers (specialized multidisciplinary palliative care services, GP, clinical specialist, nurse, caretaker/home carer, spiritual caregiver, physio-, occupational-, speech therapist).

*End-of-life care *was measured in terms of end-of-life care goals i.e. main treatment aim (cure, prolonging life, or palliative/comfort/supportive care), specific potentially life-prolonging and palliative treatments received, and physical, psychosocial and/or spiritual content of care [1,2]. Other end-of-life practices measured were the absence/presence of continuous deep sedation until death of the patient, and of medical end-of-life decisions with possible life-shortening effect i.e. (1) non-treatment decisions; (2) alleviation of pain or other symptoms, in dosages which are large enough to include the hastening of death as a possible or certain side-effect; and (3) physician-assisted dying (including euthanasia, physician-assisted suicide, and life-ending without the patient's explicit request). This categorisation was based on a pre-existing internationally validated framework [27,28,49].

### Measurement instrument

To develop the registration form for this study, we selected instruments used in other (Dutch) retrospective and quantitative studies whenever possible [17,27,28,50-54]. In case a specific concept could not be measured with an existing instrument, questions were developed on the basis of relevant literature and in dialogue with the Belgian Scientific Institute of Public Health (coordinating the Sentinel Network of GPs) and with a counselling project Advisory Board consisting of 5 MDs (of which 4 GPs), a palliative care physician, 2 psychologists, a medical sociologist, a health scientist, an anthropologist and a representative from a home care nursing organisation. We also copied questions from the official death certificates in Belgium, in order to optimize comparison afterwards.

Because Belgium consists of two large regions in which Dutch or French is spoken, the instrument was first developed in Dutch and then translated into French via forward-backward procedure.

The standardized weekly registration form is shown in Additional File 1 in English (forward translation from the Dutch version). The first part A concerned several patient- and residence characteristics and was filled in for all patients who met the primary inclusion criteria. Questions from Part A based on official death certificates were Q2, Q3, Q4, Q5, Q8, Q10, Q11 and Q12. Question 17 was taken from previously designed questionnaires in Belgium and the Netherlands [27,28,45]. Questions designed in dialogue with the Scientific Institute of Public Health and the counselling Advisory Board were Q6, Q7, Q9, Q13, Q14, Q15 and Q16.

For all patients who had died non-suddenly, the GPs registered part B consisting of: places of care and wishes for place of death, the caregivers involved during the last three months of life, the (goals of) end-of-life care and medical decision-making. Several aspects of the medical care provided were measured using three separate time frames: last week, second to fourth week and second to third month before death. Questions from Part B based on previously designed questionnaires in Belgium and the Netherlands [17,27,28,50-54] were Q3, Q4, Q5 (partly), Q6 and Q9 until Q16. Literature concerning life-sustaining therapies and indicators of aggressive and palliative care [11,21,55-58] additionally guided the development of Q5. Questions designed in the counselling Advisory Board were Q1, Q2, Q7 and Q8.

The quantitative registration instrument implied that all items had closed-ended response options. Also, in order to avoid the confounding effect that positive or negative connotations of several terms may or may not have (for example euthanasia or terminal sedation), we only used wording that describes the actual medical practices.

Finally, GPs were sent accompanying instructions at the beginning of each year, clearly stating the inclusion criteria of the study, and clarifying the manner in which the questions concerning cause of death and care setting trajectory needed to be filled in.

### Ethical considerations

The Scientific Institute of Public Health, co-ordinating the Belgian Sentinel Network of GPs, asks the GPs to give written informed consent at the beginning of each year, after being fully informed about the objectives and method of the research themes. The registration of deaths is part of this procedure.

Furthermore, strict procedures regarding patient anonymity are employed [59]. Every patient that is registered within the network receives an anonymous reference from the GP him/herself. An independent "Trust Service Provider" is responsible for supplementary anonymisation of patient information i.e. the patient's date of birth is registered by the GP but replaced with the patient's age before data-entry, and postal code of habitual residence is transformed into more aggregate indicators such as province and region of care. Concerning the GPs' identity, all his/her identification codes are replaced in the data files with anonymous codes during data cleaning.

The protocol of the present study was approved by the Ethical Review Board of Brussels University Hospital of the Vrije Universiteit Brussel (2004).

### Pilot study

The feasibility of the study design and the measurement instruments have been tested in an extensive pilot study of 3 months in 2004 [60]. The pilot study was also intended to estimate the number of non-sudden deaths that GPs would register per year, and the statistical power for the analyses of the main study in 2005 and 2006. The physicians were instructed to register all deaths of patients, aged one year or older, on a continuous basis during the months of inclusion (13 consecutive weeks), as well as the last death before this period.

In 2004, 176 GP practices reported one or more deaths. In total 502 deaths were reported, of which 466 were part of the GPs' practices. Patients who were not part of the GPs' practices (whose death was certified by the GP) were excluded. Sixty-eight percent (n = 318) of this group died during the inclusion period, and 62% (n = 214) of the latter group had died non-suddenly.

On the basis of this pilot study, the registration instrument was slightly adapted. It was estimated that 1310 patients who were part of the GPs' practices would be registered in 2005 by the 181 participating practices, and that 890 of these patients would have died non-suddenly.

### Data management and plan of statistical analysis

Several control measures are used to ensure data quality and to limit missing data. Data are entered weekly using a dbase-based programme. Range and skip checks prevent key-punching errors. Automatic follow-up of missing data for key variables and a consistency check during data-entry with the possibility of contacting GPs by phone, improve data quality. Key-punch errors are corrected via double data-entry.

Data cleaning and data analyses for this study are performed using SPSS14.0 (SPPS Inc). During data cleaning, no manual changes are made. Instead, all operations are stored via SPPS syntax-files to create a working data file. Databases are stored with a central data manager at the Scientific Institute of Public Health. To answer the first two descriptive objectives of the study concerning the places of care, and medical care provided, we plan to use mainly descriptive statistics i.e. valid percentages and 95% Confidence Intervals. To study the characteristics associated with these outcome measures (objective 3), we will use multivariate logistic regression analyses.

To verify if a representative population-based sample of deaths can be obtained via this representative sample of GPs, we will evaluate whether the deaths registered by the Sentinel Network of GPs are comparable in terms of age, sex, place of death and cause of death, to the deaths occurring within the general population (using bivariate and multivariate statistics). Since there are no recent mortality statistics available for the Southern Region of Belgium, the comparison will be made only for the data from the Northern and Brussels Capital Region (66% of the total population).

## Discussion

The objectives of this nationwide study are to describe end-of-life care provided to patients who had died non-suddenly in Belgium, and to investigate patient, disease and healthcare characteristics associated with variations in end-of-life care. To realize these objectives, a mortality follow-back study with data collection via the Belgian Sentinel Network of General Practitioners was set up.

From the participating GP practices, a total of 1385 patients had died between January 1^st ^and December 31^st ^2005. Thirty-four percent was judged to have died suddenly and totally unexpectedly. From January through December 2006, a total of 1305 patients had died, of which 37% suddenly and totally unexpectedly. Using the Sentinel Network of GPs, we obtained a percentage of sudden deaths, analogue to that reported in previous nationwide studies on end-of-life decisions based on the "death certificate sampling" design [28]. The first results of the SENTI-MELC study are expected in 2007 [61].

This study has several potential strengths as well as limitations associated with the use of an existing surveillance network of GPs in general, and with the specific design of the study.

### Strengths

The Belgian Sentinel Network of General Practitioners is representative of all GPs in Belgium, has a long tradition in scientific research (operational since 1979), is very flexible in terms of acceptability of new registrations, and is very stable in terms of participating GPs. The GPs are highly motivated to monitor various health-related problems over long and repeated periods, thus have not been selected on the basis of a specific interest in end-of-life research. Because detailed information concerning the care provided is not always available from the patients' medical files, a registration directly with GPs has an important surplus value. Since general practice is highly accessible in Belgium, a representative sample of the entire population can be monitored. Comparisons between the national vital statistics and the (age- and sex-specific) incidences of stroke mortality and suicide registered via this Sentinel Network, and between several national and international cancer registries and the cancer incidence registered via this Sentinel Network, have shown that the Sentinel Network can provide information representative for the whole population of Belgium [38,62-64].

A specific strength of this mortality follow-back study itself is that retrospective recall bias found in other retrospective research designs [28,35], will be limited, because of the weekly registrations, leaving little time between death and registration. Also, identification of non-sudden deaths as denominator is an advantage over other prospective and retrospective designs that have been criticized for selecting patients solely on the basis of diagnose or cause of death. Not all patients with a cancer diagnose, for example, also die of cancer or receive care with an end-of-life intent [34,47,65]. By avoiding to include patients that died suddenly and unexpectedly, we will be able to study care that was truly delivered in the context of a dying process. On the basis of literature, we judged the last three months of life as a relevant study period [34,46-48].

### Weaknesses

The surveillance system also has several weaknesses. Because in Belgium, there are no patient lists per practice, the population denominator (the "sentinel population") is not precisely defined and has to be estimated on the basis of annual total number of patient encounters in the participating practices [40]. Also, the registration form is to be kept simple, and time-consuming questions should be avoided in a surveillance system. Consequently, in-depth study of some aspects of care is generally not possible via this type of registration research [32,43].

Possible weaknesses of the mortality study are the retrospective data collection approach making reconstruction of all care provided in the final three months of life difficult [35] and the reliance on GPs to report care and decisions at the end of life, including care delivered to patients in hospitals or decisions taken by hospital physicians. An underestimation of specific types of care provided or decisions taken is thus possible. The extent of this problem will be studied in detail.

### Opportunities for further research

Firstly, while data gathering with the Sentinel Network in 2005–2006 focused on the processes of care delivered, a similar data gathering with the same network has been set up, aimed at investigating outcomes of quality of end-of-life care and at developing quality indicators. Data will be gathered from 2007 until 2009. Secondly, because data can be gathered over time, we will, in the long-run, evaluate the monitoring potential of this instrument. The results could potentially serve as baseline data to monitor end-of-life care over time. Thirdly, a study with Sentinel Networks creates important opportunities for international comparative research. Most European countries have at least one sentinel network of GPs [32,33,37]. The death registration that was started up in Belgium in 2005 has also been implemented in the Netherlands, and other European countries (Italy, Switzerland, Spain) are currently investigating if analogue studies are possible.

In conclusion, societal knowledge concerning the end of life has been limited to place and causes of death. Additionally, and for the first time in Belgium, with the SENTI-MELC study, we will build a public health database on how people are dying and what care they are receiving at the end of life. It will be possible to make practice and healthcare policy recommendations on the basis of this information.

## Abbreviations

Sentinel network Monitoring End-of-Life Care (SENTI-MELC)

General practitioner (GP)

## Competing interests

The author(s) declare that they have no competing interests.

## Authors' contributions

LVDB, VVC, RD, SB and LD were involved in the conception and design of the study. LVDB, NB and KD gathered the data. Statistical analyses are carried out by LVDB and critically revised by RD, NB, JB and LD. The manuscript was drafted by LVDB with critical input from all other authors. LD was the project supervisor. VVC and NB are part of the coordinating centre of the Sentinel GP Network. All authors read, revised and approved the final manuscript.

## Pre-publication history

The pre-publication history for this paper can be accessed here:



## Supplementary Material

Additional File 1 The standardized weekly registration form of the SENTI-MELC study 2005–2006 Belgium.
