# Supplementary material for: Nationwide monitoring of end-of-life care via the Sentinel Network of General Practitioners in Belgium: the research protocol of the SENTI-MELC study
Source: BMC Palliat Care. 2007 Oct 8;6:6. doi: 10.1186/1472-684X-6-6 (PMC2222051; doi:10.1186/1472-684X-6-6)
Supplement: Additional File 1 — The standardized weekly registration form of the SENTI-MELC study 2005–2006 Belgium. [file 1472-684X-6-6-S1.pdf]

FIGURE 1 The standardized weekly registration form of the SENTI-MELC study 2005-2006 Belgium

© Vrije Universiteit Brussel – End-of-Life Care Research Group

Please contact Prof.dr. Luc Deliens or Lieve Van den Block if you want to use this instrument, in full or individual items, or if you want to adapt it to your local circumstances

Status of the translation: translated from Dutch to English, no back-translation

REGISTRATION OF ALL DEATHS OF PATIENTS (AGED 1 YEAR OR OLDER)

A

1. Your reference (e.g. initials) : ....
2. Date of birth : . . / . . / . . . .
3. Date of death : . . / . . / 2 0 0 5
4. Gender
   - ☐ Male
   - ☐ Female
5. Postal code of patient's usual place of residence ☐☐☐☐
6. Did the patient have a regular partner at the time of death ?
   - ☐ yes
   - ☐ no
7. Did the patient live at home or with family (usual place of residence) ?
   - ☐ yes, alone
   - ☐ yes, with other people (state exact number) : . .
   - ☐ no
8. Patient's education (highest completed level of education or certificate)
   - ☐ primary education or less
   - ☐ lower secondary education
   - ☐ higher secondary education
   - ☐ higher education, university
9. Your estimate of the financial / material status of the patient's nuclear family
   - ☐ very low
   - ☐ rather low
   - ☐ average
   - ☐ fairly high
   - ☐ very high
10. Place of death
    - ☐ home
    - ☐ hospital
    - ☐ care home
    - ☐ public road
    - ☐ at work
    - ☐ other (namely) : ....
      ....
11. Nature of death
    - ☐ natural causes (**incl.** euthanasia)
    - ☐ traffic accident
    - ☐ other accident
    - ☐ suicide
    - ☐ murder
    - ☐ is under investigation
    - ☐ cannot be determined
12. Cause of death * : Illness or disorder that was the direct cause of death : State below under (a) the logical association of the illnesses/disorders that resulted in the immediate cause of death. If more than one illness, state the illness that was the "original cause of death" last. *This is not the way in which the patient died, e.g. heart failure, syncope, etc. ... but the illness, the trauma or the complication that caused the death. Please mention one cause per line.

    (a) ....
    Caused by : (b) ....
    Caused by : (c) ....
    Caused by : (d) ....

    see instructions for examples
13. Did you certify the death of this patient yourself ?
    - ☐ yes
    - ☐ no
14. Was this patient part of your own practice (or group practice) ?
    - ☐ yes
    - ☐ no

If "NO" to question 14, do not answer any further questions on this form

15. For approximately how long was this patient part of your practice ?
    - ☐ < 1 month
    - ☐ 1 – 3 months
    - ☐ 4 – 6 months
    - ☐ 7 – 12 months
    - ☐ 1 – 5 years
    - ☐ > 5 years
16. Did this patient have a medical file at your practice at the time of death ?
    - ☐ yes, a traditional medical file
    - ☐ yes, a global medical file (GMF)
    - ☐ no
17. **Was death sudden <u>and</u> totally unexpected ?**
    - ☐ yes
    - ☐ no

If "YES" to question 17, do not answer any further questions on this form

B

1. The **place of death** of the patient and **place(s) of residence** during the **last 3 months (=90 days) before death**, as well as the duration of stay in **days** (approximately if not precisely known). If the patient remained in the same place until death, only fill in Place 1.

see instructions for examples	at home or living with familly (incl. service flat)	care home: home for elderly persons / nursing home	hospital (**excl.** pall care unit and **excl.** nursing home unit in hospital)	palliative care unit (hospital)	somewhere else **(please specify)**
**1** place of **death** and duration of stay	☐ . . . . days	☐ . . . . days	☐ . . . . days	☐ . . . . days	☐ . . . . days
**2** previous place of **residence** and duration of stay	☐ . . . . days	☐ . . . . days	☐ . . . . days	☐ . . . . days	☐ . . . . days
**3** previous place of **residence** and duration of stay	☐ . . . . days	☐ . . . . days	☐ . . . . days	☐ . . . . days	☐ . . . . days
**4** previous place of **residence** and duration of stay	☐ . . . . days	☐ . . . . days	☐ . . . . days	☐ . . . . days	☐ . . . . days

last 3 months before death

2. Were you informed (verbally or in writing) of the patient's preference regarding their place of death ?
(More than one answer can be given)
☐ yes ☐ by the patient him/herself
☐ by the patient's family or significant other
☐ by a colleague physician
☐ by a (member of a) specialist palliative care team
☐ the information was written in the patient's medical file
☐ other (namely) : ....
If **YES**, where did this patient prefer to die ?
☐ at home or living with family (incl. service flat)
☐ in a care home: home for elderly persons / nursing home
☐ in hospital (**excl.** palliative care unit, and **excl.** nursing home unit in hospital)
☐ palliative care unit (hospital)
☐ somewhere else (namely) : ....
☐ no

3. How often (on average) did you have **contact (**consultations, home visits, **excl**. telephone contact) with the patient or with significant others regarding the patient ?

**last week before death**	**2nd to 4th week before death**	**2nd and 3rd month before death**
. . . x per **week**	. . . x per **week**	. . . x per **month**

4. What was the **main goal** of this patient's treatment ?
(More than one answer can be given)

	**last week before death**	**2nd to 4th week before death**	**2nd and 3rd month before death**
cure	☐	☐	☐
prolonging life	☐	☐	☐
comfort / palliation	☐	☐	☐
unknown	☐	☐	☐

5. Indicate what treatment/aids the patient received
(More than one answer can be given)

	**last week before death**	**2nd to 4th week before death**	**2nd and 3rd month before death**
A unknown	☐	☐	☐
B chemotherapy or radiotherapy (curative or life-prolonging)	☐	☐	☐
C artificial food administration	☐	☐	☐
D artificial fluid administration	☐	☐	☐
E blood transfusion	☐	☐	☐
F artificial respiration	☐	☐	☐
G cardiopulmonary resuscitation (CPR)	☐	☐	☐
H dialysis	☐	☐	☐
I antibiotics	☐	☐	☐
J vasopressors	☐	☐	☐
K specific palliative medication (e.g. analgesics, anti-depressants, sedatives, corticoids, anti-emetics, laxatives, anti-hiccough/ anti-irritation, ...)	☐	☐	☐
L comfort care materials (e.g. for incontinence or wound/stoma care,...)	☐	☐	☐
M general comfort aids (e.g. mattresses, hospital bed, commode,...)	☐	☐	☐
N none of the above	☐	☐	☐

6. Circle the extent to which the care provided (by you and/or other caregivers) was directed at :

1=not or to a very small extent **5**=to a very large extent **?**=unknown

	**last week before death**	**2nd to 4th week before death**	**2nd and 3rd month before death**
treatment / care for physical problems	1 2 3 4 5 ?	1 2 3 4 5 ?	1 2 3 4 5 ?
psychosocial care	1 2 3 4 5 ?	1 2 3 4 5 ?	1 2 3 4 5 ?
spiritual care (existential, religious, etc)	1 2 3 4 5 ?	1 2 3 4 5 ?	1 2 3 4 5 ?

© Vrije Universiteit Brussel – End-of-Life Care Research Group
Draft translation

7. How often were the following persons/disciplines actively involved in providing care for this patient during the last 3 months of life?

	not at all	sometimes	often	unknown
you yourself	☐	☐	☐	☐
clinical specialist	☐	☐	☐	☐
informal caregiver (e.g. partner, child, sister, friend, …)	☐	☐	☐	☐
nurse	☐	☐	☐	☐
carer / home carer / geriatric assistant (**excl.** nurse)	☐	☐	☐	☐
chaplain / pastor / non-religuous counsellor	☐	☐	☐	☐
physiotherapist / occupational therapist / speech therapist	☐	☐	☐	☐
other (namely) :	☐	☐	☐	☐

8. Which **specialist palliative care initiatives** were brought into action in the last 3 months of this patient's life?
(More than one answer can be given)
- ☐ 1 palliative homecare team
- ☐ 2 mobile palliative care support team in a hospital
- ☐ 3 palliative care unit (hospital)
- ☐ 4 reference persons for palliative care in a care home (co-ordinating and advisory physician and/or reference nurse)
- ☐ 5 LEIF or EOL- physician (End-of life Information Forum) (LEIF or EOL physicians provide support and advice to colleague physicians concerning issues related to euthanasia, medical end-of-life decisions, and the possibilities of palliative care in Belgium)
- ☐ 6 palliative day (care) centre
- ☐ 7 other (namely) : ....
- ☐ 8 none
- ☐ 9 unknown

9. Was the patient kept continuously in deep sedation or coma until death by means of e.g. benzodiazepines or barbiturates?
- ☐ yes, and food and fluids were not artificially administered
- ☐ yes, and food and fluids were artificially administered
- ☐ no

The following questions concern medical decisions, with a (potentially) life-shortening effect for the patients

10. Did you or a colleague physician take one or more of the following acts (or ensure that one of them was taken), taking into account the probability or certainty that this act would hasten the end of the patient's life? (**Please answer both questions 10a and 10b**):

10a. withholding a treatment or withdrawing a treatment (incl. artificial administration of food and/or fluids) ? ☐ yes ☐ no

10b. intensifying the alleviation of pain and/or symptoms using a drug? ☐ yes, ➔**go to question 11** ☐ non,➔**go to question 12**

11. Was hastening the end of life partly the intention of the act indicated in question **10b** ? ☐ yes ☐ no

12. Was death caused by withholding a treatment or withdrawing a treatment (incl. artificial administration of food and/or fluids), which you or a colleague physician decided to take with the explicit intention of hastening the end of life ?
- ☐ yes
- ☐ no

13. Was death caused by the use of a drug prescribed, supplied or administered by you or a colleague physican with the explicit intention of hastening the end of life (or of enabling the patient to end his or her own life) ?
- ☐ yes
- ☐ no

If yes, who administered this drug (= introduced it into the body) ?
(More than one answer can be given)
- ☐ the patient
- ☐ you or a colleague physician
- ☐ nursing staff/other caregivers
- ☐ other (namely) : ....

The following questions relate to the LAST "YES" in answer to questions 10a to 13

If you answered "no" to all parts of these questions, do not answer any further questions on this form

14. Did you or a colleague physician discuss with the patient the (possible) hastening of the end of life as a result of the (last-mentioned) act?
- ☐ yes, at the time of performing the act or shortly before
- ☐ yes, some time beforehand
- ☐ no, no discussion

15. Was the decision concerning the (last-mentioned) act made upon an explicit request of the patient?
- ☐ yes
- ☐ no, but the patient had expressed a wish
- ☐ no, and the patient had never expressed a wish

16. Did you consider the patient capable to assess his/her situation and to make a decision about it adequately ?
- ☐ yes
- ☐ no, not fully capable
- ☐ no, not capable at all

© Vrije Universiteit Brussel – End-of-Life Care Research Group
Draft translation
